# A new era for Type 2 diabetes genetics

**DOI:** 10.1111/j.1464-5491.2007.02274.x

**Published:** 2007-11

**Authors:** E. Zeggini

**Affiliations:** The Wellcome Trust Centre for Human Genetics, University of Oxford Oxford, UK

**Keywords:** genome-wide association, Type 2 diabetes

## Abstract

Diabet. Med. (2007)

## Introduction

The field of complex disease genetics has witnessed rapid progress over the past few months. The advent of genome-wide association scans (GWAS) has mediated a change in gear and the Type 2 diabetes (T2D) research community has set an unprecedented record with five genome-wide association studies published since February 2007 [1–6], increasing the number of confirmed Type 2 diabetes susceptibility loci from three (*PPARG*, *KCNJ11*, *TCF7L2*) to nine (with the addition of *CDKAL1*, *CDKN2A*/*B*, *IGF2BP2*, *HHEX*/*IDE*, *FTO* and *SLC30A8*). These studies have increased our understanding of the genetic aetiology of T2D and provided invaluable insights into the way genetic studies should be conducted. This review aims to summarize the major findings distilled from the dawn of this new era in T2D genetics.

The feasibility of carrying out GWAS has been eagerly anticipated in the field of T2D genetics for some time. Several years of mostly unsuccessful gene hunting led to the realization that carefully designed and conducted, large-scale, biology-agnostic studies would be a necessary addition to the geneticist's arsenal. Whole genome linkage scans, candidate gene-focused association studies and region-specific fine-mapping approaches had dominated the literature over the past few years. However, only three genes (*PPARG*, *KCNJ11* and, more recently, *TCF7L2*) had been established as T2D susceptibility loci [7–9]. Several reasons can account for the paucity of success stories in the scientific literature, but the biggest culprits stand out as insufficient power, over-interpretation of results and candidacy assessments based on incomplete knowledge of T2D aetiopathology. Lack of power in genetic association studies could be ascribed to a variety of factors, including insufficient sample sizes to detect the modest effects conferred by susceptibility variants, as well as an incomplete understanding of human genetic variation. Indeed, it was not unusual for gene mapping studies to examine only a handful of variants, or even a single variant, per gene (most likely in a coding portion of the transcribed unit).

## Setting the stage for genome-wide studies

Evolution of our understanding of the human genome has been made possible by the development of high-throughput genotyping technologies, enabling endeavours such as the International HapMap project [10]. The HapMap has given us insights into common variation genome-wide by cataloguing single nucleotide polymorphisms (SNPs), their frequencies and pairwise linkage disequilibrium (LD) relationships in samples from four populations. In addition, emerging evidence of the putatively important role of non-protein-coding DNA (as exemplified by conserved non-coding regions and microRNAs) [11,12] have dictated a shift in focus from gene-centric to genome-wide examination of human variation. High-throughput genotyping technologies have now become an affordable reality, allowing the genome-wide profiling of large-scale, well-phenotyped samples, which are also becoming increasingly available (and which will be necessary to detect the moderate effects that T2D susceptibility variants are expected to confer).

Two main commercially available platforms for genome-wide SNP genotyping have been used by the five published T2D association scans to date: the Affymetrix 500k and the Illumina HumanHap300 arrays. The fixed SNP content on these chips has been differently selected. Variants on the Affymetrix 500k platform have been selected to provide an approximately random distribution of SNPs across the genome, without taking LD patterns into account. In contrast, the Illumina HumanHap300 chip contains approximately 317k SNPs, specifically selected to tag common variation [as defined by a minor allele frequency (MAF) > 5%], making use of pairwise correlation information between HapMap phase I SNPs in the European-descent CEU sample. Pairwise evaluations of the proportion of variation captured on the basis of HapMap phase II indicate that both the Affymetrix and Illumina platforms provide good coverage of common SNPs genome-wide in a single experiment (65% and 75%, respectively) [13,14]. Each platform balances different advantages and limitations. For example, the Affymetrix chip captures a smaller proportion of common variation genome-wide, but is not population specific and has inbuilt redundancy (as ascribed by the LD-agnostic design), thus suffering a smaller decrease in performance with realistic genotype failure rates. Neither platform used by the five reported T2D genome-wide association scans is exhaustive (for example, rare SNPs and structural variation are under-represented), but new generation genome-wide arrays have been designed to be more comprehensive.

## Four months, five scans, six new T2D loci

The field of T2D has been at the forefront of genetic association studies for some time, with a recent history of successful large-scale well-designed candidate gene studies and an emerging strong collaborative ethos (for example, the International T2D 1q Consortium, formed to carry out fine mapping of the chromosome 1q linkage peak in eight populations). It remains impressive that five genome-wide association scans for the disease have been published in the space of less than 4 months. These have led to the identification of six novel T2D susceptibility loci.

The five T2D genome-wide association scans published to date have followed a range of study designs. Sladek *et al*. [5], the first scan to be published, employed two Illumina platforms: the HumanHap300 chip and the Infinium Human1 array, which probes approximately 100k gene-centred SNPs. Using a moderately sized sample set in their primary scan, Sladek *et al*. focused on French T2D cases with at least one affected first-degree relative (thus enriching for susceptibility allele frequency), young age at onset and a body mass index (BMI) less than 30 kg/m^2^. Upon replication of their most significant findings in a further set of 5511 individuals, they were the first to report the now confirmed *SLC30A8* and *HHEX*/*IDE* genes as novel T2D susceptibility loci. Further replicating loci in this French study (*EXT2-ALX4* and *LOC387761*) have not met confirmation by the four subsequent scans.

Steinthorsdottir *et al*. [6] also used the Illumina HumanHap300 array, to genotype a total of 6674 T2D case and control subjects from Iceland, confirming the *SLC30A8* and *HHEX*/*IDE* findings. Extensive replication studies in samples from five different populations (Denmark, Philadelphia, the Netherlands, Hong Kong and West Africa) led to the identification of *CDKAL1* as a T2D susceptibility locus. This novel T2D gene was simultaneously reported by the Steinthorsdottir *et al*. [6], UK-based [1,2], Scott *et al*. [4] and Diabetes Genetics Initiative (DGI) [3] studies.

In an unprecedented collaboration, the latter three studies joined forces to combine the findings of both their primary scans and follow-up endeavours (leading to a total of 32 554 samples), identifying *CDKAL1*, *CDKN2A*/*B*, *IGF2BP2* and *FTO* as novel T2D susceptibility loci and confirming the *SLC30A8* and *HHEX*/*IDE* associations.

Individually, the UK-based T2D GWAS was carried out as part of the Wellcome Trust Case Control Consortium (WTCCC) study, which included approximately 14 000 cases of seven common diseases (~2000 of which were T2D cases, partly enriched for younger age at onset and for family history of the disease) and 3000 shared control subjects, genotyped on the Affymetrix 500k platform [1]. Interesting findings were subsequently followed up in at least 9103 T2D case and control subjects [2,15]. Scott *et al*. [4] genotyped 2335 Finnish T2D case and control individuals on the HumanHap300 array and then analysed imputed genotypes HapMap-wide. Imputation involves the use of phased HapMap CEU data to infer genotypes at untyped positions. Second stage genotyping was performed in 2473 further individuals. The DGI employed the Affymetrix 500k chip to genotype 3077 individuals from Finland and Sweden in a combination of population- and family-based samples [3]. Replication studies involved 10 850 T2D case and control subjects.

Tables 1 and 2 summarize the main characteristics of each primary scan and associated follow-up experiments, respectively. Upholding the gold standard in association studies, all five genome-wide association scans included a replication stage, where the robustness of identified associations was tested.

**Table 1:** Primary scan characteristics

Genome-wide association scan	Platform	Cases (*n*)	Control subjects (*n*)	Population	T2D phenotype
Sladek *et al*. [5]	Illumina HumanHap300 Infinium Human1	661	614	France	Family history of T2D, AAO < 45 years, BMI < 30 kg/m^2^
WTCCC [1]	Affymetrix 500k	1924	2938	UK	Partial enrichment for family history ofT2D, AAO < 65 years
Scott *et al*. [4]	Illumina HumanHap300	1161	1174	Finland	Partial enrichment for family history
DGI [3]	Affymetrix 500k	1464	1467	Finland, Sweden	Partial enrichment for family history
Steinthorsdottir *et al*. [6]	Illumina HumanHap300	1399	5275	Iceland	No specific enrichment for family history, young AAO or BMI

AAO, age at onset; BMI, body mass index; DGI, Diabetes Genetics Initiative; T2D, Type 2 diabetes; WTCCC, Wellcome Trust Case Control Consortium.

**Table 2:** Stage 2/replication study characteristics

Genome-wide association follow-up	Cases (*n*)	Control subjects (*n*)	Population	T2D phenotype	Confirmed T2D loci reported
Sladek *et al*. [5]	2617	2894	France	BMI < 35 kg/m^2^	*TCF7L2*, *SLC30A8*, *HHEX/IDE*
Zeggini *et al*.*[2]	3757	5346	UK	Partial enrichment for AAO < 45 years	*TCF7L2*, *CDKAL1*, *CDKN2A*/*B*, *HHEX*/*IDE*, *SLC30A8*, *IGF2BP2*, *FTO*, *PPARG*, KCNJ11*
Scott *et al*.*[1]	1215	1258	Finland	No specific enrichment	*TCF7L2*, *CDKAL1*, *CDKN2A*/*B*, *HHEX*/*IDE*, *SLC30A8*, *IGF2BP2*, *FTO*, *PPARG*, KCNJ11*
DGI*[4]	5065	5785	European ancestry†	No specific enrichment	*TCF7L2*, *CDKAL1*, *CDKN2A*/*B*, *HHEX*/*IDE*, *SLC30A8*, *IGF2BP2*, *FTO*, *PPARG*, KCNJ11*
Steinthorsdottir *et al*. [6]	3826	12562	European ancestry‡	No specific enrichment	*TCF7L2*, *CDKAL1*, *SLC30A8*, *HHEX*/*IDE*
	1457	986	Hong Kong	Partial enrichment for AAO < 40 years	
	865	1106	West Africa	Family history of T2D	

* The Zeggini *et al*. [2], Scott *et al*. [4] and DGI [3] replication studies included meta-analysis of primary scan and stage 2 datasets from each of the three studies.

† From Sweden, Poland and the USA.

‡ From Denmark, Philadelphia and the Netherlands.

AAO, age at onset; BMI, body mass index; DGI, Diabetes Genetics Initiative; T2D, Type 2 diabetes.

## Characteristics of the six robustly replicating genome-wide association signals

Indirect LD mapping of disease variants is an inherent characteristic of genome-wide association scans using currently available defined-content SNP chips. As such, the five published T2D GWAS have identified six novel, robustly replicating signals within the limits of resolution afforded by the respective arrays employed. This makes unequivocal identification of T2D disease-predisposing genes difficult. Further fine mapping (by genotyping a dense map of polymorphisms in diverse populations) and/or extensive resequencing efforts will be necessary before a single gene can be declared as most likely to be causal. Of the six confirmed T2D association signals, four appear to cluster within specific genes (*CDKAL1*, *SLC30A8*, *IGF2BP2*, *FTO*) and two extend across larger regions (near *CDKN2A*/*B* on chromosome 9 and near *HHEX*/*IDE* on chromosome 10).

All six replicating associations were observed at common sequence variants, i.e. SNPs with relatively high minor allele frequencies. This is, however, not unexpected, given SNP chip content and association power limitations brought about by the sample sizes employed (Table 1). Although we cannot exhaustively assess the genetic architecture of T2D before examining variants across the full spectrum of allele frequencies in sufficiently powered studies, it has now become clear that multiple common variants are certainly part of the picture and play a role in increasing susceptibility to T2D.

Elucidating the genetic model under which known disease variants operate can prove a helpful guide for future studies. With the possible exception of *CDKAL1*, all novel T2D loci currently appear to follow an additive mode of inheritance, reflecting a linear change of disease risk with each extra allele copy carried. This observation could, however, prove to be an artefact of allelic association (LD) and the fact that the truly causal variant remains to be identified.

The per-allele effect sizes of all six loci were found to be modest, with odds ratios approximately ranging from 1.1 to 1.2. This is a sobering observation that highlights the need for large sample sizes if further loci are to be identified at genome-wide levels of significance (for example, over 11 500 cases and an equal number of control subjects would be necessary to detect an effect size of 1.15 with 80% power at *P* = 10^−7^ for a common SNP with 20% minor allele frequency).

Phenotype definition can also prove crucial in determining power to discover disease genes. The *FTO* signal, for instance, was identified and quickly replicated as part of the UK genome-wide association study [1,2], but was not independently observed in any of the other scans. It subsequently transpired that *FTO* exerts its effect on T2D risk through adiposity and has since been established as an obesity gene [15]. Although failure by four of the five scans to identify *FTO* as a diabetes gene may be ascribed to sample size differences, variable phenotype definition (for example, in Sladek *et al*. [5], Tables 1 and 2) and case-control matching for BMI (DGI scan [3]) could have led to significant dilution of the signal, rendering it undetectable.

## Insights into T2D aetiology

Genome-wide association studies do not rely on gene candidacy assessment and offer the hope of gaining insights into disease aetiopathology. The five T2D GWAS have achieved just that. But why had the six new T2D loci escaped the radar of traditional approaches and what insights into T2D aetiology have we gained?

The *HHEX*/*IDE* signal resides under a well-replicated T2D peak of linkage (a few megabases away from *TCF7L2*, on chromosome 10q). However, the associated variants do not possess characteristics (in terms of allele frequency and effect size) that would explain the linkage signal observed. Nevertheless, *IDE* (coding for insulin degrading enzyme) has been studied as a T2D candidate gene in the past, giving rise to inconsistent associations [16–18]. The *IDE* story highlights how important comprehensive capture of sequence variation is, before any gene can be discounted. *HHEX* (homeobox, haematopoietically expressed) encodes a transcription factor important for pancreatic development [19,20] and (retrospectively) constitutes a strong candidate. The chr10q T2D signal in this region also extends over *KIF11*, encoding kinesin interacting factor. Recent data suggest that T2D risk variants within this region are associated with reduced pancreatic β-cell function [21].

*IGF2BP2*, coding for insulin-like growth factor 2 mRNA binding protein 2, resides in the vicinity of the chr3q linkage peak for T2D and related traits and, although a good candidate, had not previously been the focus of a T2D genetic association study. The genome-wide association signal spans the promoter and first two exons of this gene. IGF2BP2 binds the 3′ UTR of the insulin-like growth factor 2 (*IGF2*) transcript and regulates *IGF2* translation [22]. IGF2 is involved in cell proliferation, differentiation and stimulation of insulin action.

The chr9p signal is idiosyncratic and will undoubtedly require extensive rounds of fine mapping and resequencing before the causal functional unit(s) can be determined. The most strongly associated SNPs reside 3′ of *CDKN2A* and *CDKN2B* in a small region with no characterized genes, delineated by recombination hotspots. Interestingly, a strong signal for heart disease (driven by SNPs independent of those implicated in T2D) also resides in this region [1,23,24]. It is possible that this overlap may reflect common aetiopathogenic pathways. *CDKN2B*and *CDKN2A*code for p15^INK4b^ and p16^INK4a^, respectively. p16^INK4a^ plays a role in pancreatic B-cell replication by inhibiting CDK4 (cyclin-dependent kinase 4) [25–27]. Evidence from murine models also demonstrates a central role in islet proliferation for both *CDKN2A* and *CDKN2B*[28,29].

The *CDKAL1*[cyclin-dependent kinase 5 (CDK5) regulatory subunit associated protein 1-like 1] signal is centred in intron 5 of the gene (highly expressed in human pancreas and skeletal muscle [2]). Although the gene product function has not yet been characterized, protein homology to CDK5 regulatory subunit-associated protein 1 indicates a role in CDK5 inhibition. CDK5 is important in the glucotoxic loss of B-cell function [30,31]. T2D susceptibility-associated variation in *CDKAL1*has been shown to confer risk through reduced insulin secretion [6,21]. Specifically, Steinthorsdottir *et al*. [6] showed that the insulin response for carriers of two copies of the risk allele was 22% lower than for heterozygotes or non-carriers.

The T2D-associated SNP in *SLC30A8* is a non-synonymous Arg→Trp variant. *SLC30A8* codes for a pancreatic islet-specific zinc transporter and is involved in insulin biosynthesis, maturation and storage by mediating zinc accumulation in intracellular insulin-containing vesicles. The *SLC30A8* T2D variant has also been found to be associated with decreased insulin secretion [6].

*FTO*, although identified through a T2D GWAS, has already been established as a robustly replicating obesity locus [15,32]. However, the mechanism through which *FTO* might exert its function remains unknown. It is widely expressed, with pronounced transcription levels in the brain [15]. Clearly, our understanding of its mode of action is still in its infancy.

## Impact on diabetes care

The five published T2D genome-wide association scans signify huge strides forward for the field and their findings are expected to make an impact on diabetes care. They offer the potential to explore new biology and develop novel treatments, following the examples of *PPARG* and *KCNJ11*, where therapeutic agents (thiazolidinediones and sulphonylureas, respectively) and genes are closely associated. There are undoubtedly many exciting lessons on diabetes biology to be learnt from the six new susceptibility loci. Overall, the scales appear to tip towards aetiological pathways involved in pancreatic B-cell development, function and regeneration. Interdisciplinary endeavours involving genetic, functional and physiological studies will now be necessary to take this new knowledge further.

The associated variants could also have diagnostic and prognostic potential. Scott *et al*. [4] calculated a fourfold variation in T2D risk between the carriers of all known risk alleles and non-carriers. However, as the SNPs have common frequencies and moderate effect sizes, their immediate diagnostic potential is reduced. Their combined predictive power is also currently too small to be clinically useful ([33]; M. N. Weedon, personal communication). It is possible that rarer, more penetrant variants will be of higher predictive value, but these remain to be identified and would be expected to affect a smaller proportion of T2D patients. In addition, the methodology by which to unearth such sequence variation is not yet optimized. However, rapid advances in resequencing technologies and analytical approaches are poised to make this possible in the near future.

## Taking the next steps forward

Replication of the major GWAS findings in diverse populations, fine mapping of the association signals and extensive resequencing of implicated genes will now form the next steps in the field of T2D genetics. But what about signals that have not yet been unearthed? Emerging imputation approaches will make a more comprehensive assessment of human genome variation possible. However, proactive collaborative efforts will be necessary in order to attain sufficient power to detect and replicate novel T2D genes. Functional and physiological studies in well-characterized cohorts will ultimately provide us with clear answers and pave the way for meaningful translation of this research into diabetes care.

## Abbreviations

BMI: body mass index
CEU: CEPH (Utah residents with ancestry from Northern and Western Europe)
DGI: Diabetes Genetics Initiative
GWAS: genome-wide association scans
LD: linkage disequilibrium
SNP: single nucleotide polymorphism
T2D: Type 2 diabetes

## Glossary

Single nucleotide polymorphism (SNP): A single nucleotide polymorphism is a single-base change in the human genome sequence. SNPs are the commonest form of human genome variation and occur every 100–300 base pairs.
Linkage disequilibrium (LD): Linkage disequilibrium or allelic association is the occurrence of alleles together on the same chromosome more often than expected based on their allele frequencies.
Untranslated region (UTR): Untranslated regions are portions of mRNA that do not code for proteins. UTRs contain sequence important for translation regulation and mRNA stability.
High-throughput genotyping: High-throughput, highly parallel genotyping assays are highly automated and allow rapid processing of a large number of samples. Automated software tools are used for the assignment of genotypes.
Case-control association study: In the case-control association study design, sequence variant frequencies are compared between a sample of affected individuals (cases) and an appropriate control group.

## References

[1] Wellcome Trust Case Control Consortium (2007). Genome-wide association study of 14 000 cases of seven common diseases and 3000 shared controls. Nature.

[2] Zeggini E, Weedon MN, Lindgren CM, Frayling TM, Elliott KS, Lango H (2007). Replication of genome-wide association signals in UK samples reveals risk loci for type 2 diabetes. Science.

[3] Diabetes Genetics Initiative of Broad Institute of Harvard and MIT, Lund University, and Novartis Institutes of BioMedical Research (2007). Genome-wide association analysis identifies loci for type 2 diabetes and triglyceride levels. Science.

[4] Scott LJ, Mohlke KL, Bonnycastle LL, Willer CJ, Li Y, Duren WL (2007). A genome-wide association study of type 2 diabetes in Finns detects multiple susceptibility variants. Science.

[5] Sladek R, Rocheleau G, Rung J, Dina C, Shen L, Serre D (2007). A genome-wide association study identifies novel risk loci for type 2 diabetes. Nature.

[6] Steinthorsdottir V, Thorleifsson G, Reynisdottir I, Benediktsson R, Jonsdottir T, Walters GB (2007). A variant in *CDKAL1* influences insulin response and risk of type 2 diabetes. Nat Genet.

[7] Altshuler D, Hirschhorn JN, Klannemark M, Lindgren CM, Vohl MC, Nemesh J (2000). The common PPARγ Pro12Ala polymorphism is associated with decreased risk of type 2 diabetes. Nat Genet.

[8] Gloyn AL, Weedon MN, Owen KR, Turner MJ, Knight BA, Hitman G (2003). Large-scale association studies of variants in genes encoding the pancreatic beta-cell K-ATP channel subunits Kir6.2 (KCNJ11) and SUR1 (ABCC8) confirm that the *KCNJ11* E23K variant is associated with increased risk of Type 2 diabetes. Diabetes.

[9] Zeggini E, McCarthy MI (2007). *TCF7L2*: the biggest story in diabetes genetics since HLA?. Diabetologia.

[10] International HapMap Consortium (2005). A haplotype map of the human genome. Nature.

[11] Bird CP, Stranger BE, Dermitzakis ET (2006). Functional variation and evolution of non-coding DNA. Curr Opin Genet Dev.

[12] Gauthier BR, Wollheim CB (2006). MicroRNAs: ‘ribo-regulators’ of glucose homeostasis. Nat Med.

[13] Barrett JC, Cardon LR (2006). Evaluating coverage of genome-wide association studies. Nat Genet.

[14] Pe’er I, de Bakker PI, Maller J, Yelensky R, Altshuler D, Daly MJ (2006). Evaluating and improving power in whole-genome association studies using fixed marker sets. Nat Genet.

[15] Frayling TM, Timpson NJ, Weedon MN, Zeggini E, Freathy RM, Lindgren CM (2007). A common variant in the FTO gene is associated with body mass index and predisposes to childhood and adult obesity. Science.

[16] Groves CJ, Wiltshire S, Smedley D, Owen KR, Frayling TM, Walker M (2003). Association and haplotype analysis of the insulin-degrading enzyme (IDE) gene, a strong positional and biological candidate for type 2 diabetes susceptibility. Diabetes.

[17] Karamohamed S, Demissie S, Volcjak J, Liu C, Heard-Costa N, Liu J (2003). Polymorphisms in the insulin-degrading enzyme gene are associated with type 2 diabetes in men from the NHLBI Framingham Heart Study. Diabetes.

[18] Florez JC, Wiltshire S, Agapakis CM, Burtt NP, de Bakker PI, Almgren P (2006). High-density haplotype structure and association testing of the insulin-degrading enzyme (IDE) gene with type 2 diabetes in 4206 people. Diabetes.

[19] Foley AC, Mercola M (2005). Heart induction by Wnt antagonists depends on the homeodomain transcription factor Hex. Genes Dev.

[20] Bort R, Martinez-Barbera JP, Beddington RS, Zaret KS (2004). Hex homeobox gene-dependent tissue positioning is required for organogenesis of the ventral pancreas. Development.

[21] Pascoe L, Tura A, Patel SK, Ibrahim IM, Ferrannini E, the RISC Consortium (2007). Common variants of the novel type 2 diabetes genes, CDKAL1 and HHEX/IDE, are associated with decreased pancreatic β-cell function. Diabetes.

[22] Nielsen J, Christiansen J, Lykke-Andersen J, Johnsen AH, Wewer UM, Nielsen FC (1999). A family of insulin-like growth factor II mRNA-binding proteins represses translation in late development. Mol Cell Biol.

[23] McPherson R, Pertsemlidis A, Kavaslar N, Stewart A, Roberts R, Cox DR (2007). A common allele on chromosome 9 associated with coronary heart disease. Science.

[24] Helgadottir A, Thorleifsson G, Manolescu A, Gretarsdottir S, Blondal T, Jonasdottir A (2007). A common variant on chromosome 9p21 affects the risk of myocardial infarction. Science.

[25] Rane SG, Dubus P, Mettus RV, Galbreath EJ, Boden G, Reddy EP (1999). Loss of Cdk4 expression causes insulin-deficient diabetes and Cdk4 activation results in beta-islet cell hyperplasia. Nat Genet.

[26] Mettus RV, Rane SG (2003). Characterization of the abnormal pancreatic development, reduced growth and infertility in Cdk4 mutant mice. Oncogene.

[27] Marzo N, Mora C, Fabregat ME, Martín J, Usac EF, Franco C (2004). Pancreatic islets from cyclin-dependent kinase 4/R24C (Cdk4) knockin mice have significantly increased beta cell mass and are physiologically functional, indicating that Cdk4 is a potential target for pancreatic beta cell mass regeneration in Type 1 diabetes. Diabetologia.

[28] Krishnamurthy J, Ramsey MR, Ligon KL, Torrice C, Koh A, Bonner-Weir S (2006). p16INK4a induces an age-dependent decline in islet regenerative potential. Nature.

[29] Moritani M, Yamasaki S, Kagami M, Suzuki T, Yamaoka T, Sano T (2005). Hypoplasia of endocrine and exocrine pancreas in homozygous transgenic TGF-β1. Mol Cell Endocrinol.

[30] Ubeda M, Rukstalis JM, Habener JF (2006). Inhibition of cyclin-dependent kinase 5 activity protects pancreatic beta cells from glucotoxicity. J Biol Chem.

[31] Wei FY, Nagashima K, Ohshima T, Saheki Y, Lu YF, Matsushita M (2005). Cdk5-dependent regulation of glucose-stimulated insulin secretion. Nat Med.

[32] Dina C, Meyre D, Gallina S, Durand E, Korner A, Jacobson P (2007). Variation in FTO contributes to childhood obesity and severe adult obesity. Nat Genet.

[33] Weedon MN, McCarthy MI, Hitman G, Walker M, Groves CJ, Zeggini E (2006). Combining information from common type 2 diabetes risk polymorphisms improves disease prediction. PLoS Med.

